# *TRIM5α*/Cyclophilin A-Modified MDBK Cells for Lentiviral-Based Gene Editing

**DOI:** 10.3390/v17070876

**Published:** 2025-06-21

**Authors:** Lijing Wo, Shuhui Qi, Yongqi Guo, Chao Sun, Xin Yin

**Affiliations:** State Key Laboratory for Animal Disease Control and Prevention, Harbin Veterinary Research Institute, Chinese Academy of Agricultural Sciences, Harbin 150069, China; huli12138@163.com (L.W.); 18734445804@163.com (S.Q.); 15581741932@163.com (Y.G.)

**Keywords:** MDBK, TRIM5α, CypA

## Abstract

The human immunodeficiency virus 1 (HIV-1)-based lentivirus has been widely used for genetic modification. However, the efficiency of lentiviral-based gene modification in Madin–Darby bovine kidney (MDBK) cells is considerably limited. In this study, we have shown that siRNA-mediated depletion of *TRIM5α*, a restriction factor in HIV-1 infection, can dramatically enhance HIV-1 infection in MDBK cells. Furthermore, we generated a doxycycline-inducible Cas9-overexpressing MDBK cell line (MDBK-iCas9) suitable for CRISPR/Cas9-mediated editing. On this basis, we created a *TRIM5α* knock-out MDBK-iCas9 cell line MDBK-iCas9*^TRIM5α−/−^* without additional genome insertions by combining sgRNA transfection and single-cell cloning. We found that MDBK-iCas9*^TRIM5α−/−^* displayed greater permissiveness to lentivirus infection compared with MDBK-WT cells. Notably, we found that treatment with the chemical compound cyclosporine A, which directly interacts with cell factor cyclophilin A (CypA), could markedly increase the infectivity of lentivirus in both MDBK-iCas9*^TRIM5α−/−^* and MDBK-WT cell lines, suggesting that *CypA* functions independently with *TRIM5α* as an inhibitor of the lentivirus in bovine cells. Therefore, combining bovine *TRIM5α* and CypA targeting could remarkably enhance lentivirus infection. In conclusion, our findings highlight a promising gene engineering strategy for bovine cells that can surmount the significant barriers to investigating the interplay between bovine viruses and their host cells.

## 1. Introduction

Madin–Darby bovine kidney (MDBK) cells, which originated from the bovine kidney, have been commonly used in virology and molecular biology research for studying bovine viral replication and virus–host interactions. Due to their susceptibility, MDBK cells have been utilized for the isolation and propagation of a wide range of bovine viruses, including bovine viral diarrhea virus (BVDV) [1], bovine herpesvirus 1 (BHV-1), bovine parainfluenza virus 3 (BPIV3), bovine respiratory syncytial virus (BRSV), and lumpy skin disease virus (LSDV) [2]. In addition to viral studies, MDBK cells are frequently employed as a model system for elucidating aspects of bovine cell biology, including cell signaling, proliferation, and differentiation. These characteristics underscore their significance as a model system for studying virus–host interactions and for biotechnological applications aimed at manipulating gene expression in bovine cells. However, MDBK cells are known to be challenging to transfect using conventional nonviral approaches [3], which has prompted the exploration of alternative gene delivery systems, such as viral-vector-based systems, including lentiviral and adeno-associated viral vectors. Among them, lentiviral vectors have been widely used because of their capacity to sustain exogenous gene existence by stably integrating their genome into the host genome in both dividing and non-dividing cells [4]. Notably, the transduction efficacy of lentivirus is extremely limited in MDBK cells, hindering this delivery strategy. Subsequently, it was found that the inhibition is mainly caused by an anti-HIV-1 host factor tripartite motif containing 5α (*TRIM5α*) in bovine cells [5,6], which limits HIV-1 virus replication by destabilizing the capsid cores of HIV-1 [7]. Therefore, the inactivation of TRIM5α in MDBK cells could enhance the susceptibility of MDBK cells to the HIV-1-based lentivirus and improve their gene modification capabilities.

TRIM5α, a member of the TRIM family, was identified as a restriction factor against HIV-1 infection [6,8,9]. It consists of three distinct domains—RING, B-box, and Coiled-coil—collectively referred to as RBCC domains [10]. It also contains a unique domain at the C-terminal segment known as the SPRY. The RING domain, located at the N-terminal of TRIM5α, plays a critical role in protein interactions and exhibits ubiquitin ligase activity. Disruption of the RING domain leads to diminished antiviral activity of TRIM5α [11]. The B-box domain can form dimers and trimers to create a hexagonal net that binds to retrovirus capsids [12]. Disruption of the B-box domain results in the loss of retroviral restriction [11]. The coiled-coil region mediates the oligomerization of the TRIM5α protein to form higher-order assemblies, enabling the protein to function as a cytosolic pattern recognition receptor that intercepts the incoming capsids of the retrovirus, including HIV-1 [13,14]. The RBCC domain motif of TRIM5α is connected to a C-terminal SPRY domain by a long linker, forming a complete TRIM5α protein. The SPRY domain enables specific recognition of the HIV-1 CA protein, triggering TRIM5α-mediated restriction [15]. Previous research has demonstrated that cellular cyclophilin A (CypA) protects HIV-1 from human TRIM5α by binding to the CA protein of HIV-1, thereby countering TRIM5α binding in CD4^+^ T cells, macrophages, and dendritic cells [16], while CypA exists in monkey cells in various forms. In new world monkey cells, such as owl monkey cells, CypA has replaced the SPRY domain of TRIM5α and formed a fusion protein TRIM5-CypA. CsA has been shown to enhance HIV-1 replication in new world monkey cell lines by disrupting the CA–CypA binding process [17,18]. Unlike this, in old world monkey cells, the protein TRIM5-CypA has been found to co-exist with the protein TRIM5α, and TRIM5α still functions as an anti-HIV factor [9,19]. However, the function of bovine CypA in HIV-1-based lentivirus replication is still unknown. Our study offers a promising strategy for gene engineering in MDBK cells, which can serve as a valuable model for deeply studying virus–cell interactions. By overcoming the limitations posed by *TRIM5α* and CypA to enhance the susceptibility of MDBK cells to lentivirus, our study opens up new possibilities for exploring the complex dynamics between bovine viruses and host cells.

## 2. Materials and Methods

### 2.1. Plasmid Construction

The following plasmids were obtained from Addgene: LentiCRISPRV_2_EGFP, LentiCRISPRV_2_Puro, and packaging plasmids pMD2.G and psPAX2. The LentiCRISPRV_2_EGFP was from David Feldser (Addgene plasmid # 82416; https://www.addgene.org/82416/, accessed on 11 April 2023) [20]. The luciferase-encoding HIV-1 lentivirus vector pNL4-3. Luc. R-E-delta Env was kindly provided by Dr. Yandong Tang at the Harbin Veterinary Research Institute of the Chinese Academy of Agricultural Sciences. To construct the doxycycline(dox)-inducible Cas9-encoding plasmid pLVX-TetOn-blasticidin-Cas9, the sequence conferring resistance to puromycin of the pLVX-TetOn-Puro vector was first replaced by the blasticidin resistance-encoding gene; next, the cDNA-encoding Cas9 was cloned into the linearized pLVX-TetOn-blasticidin vector with an infusion enzyme. To generate a *TRIM5α*-encoding pCAGGS plasmid, a cDNA-encoding *TRIM5α* was cloned into the linearized pCAGGS-HA vector with an infusion enzyme. To generate a bovine *TRIM5α*-targeting sgRNA-encoding plasmid, three single-guide RNAs designed using an online CRISPR resource (https://crispr.dbcls.jp/, accessed on 15 June 2023) that directly targeted the genomic DNA-encoding bovine *TRIM5α* were annealed and cloned into the LentiCRISPRV_2_EGFP following the Zhang Lab protocol for the generation of the *TRIM5α* knock-out cell line. To generate a bovine *NCL*- and *PPIA*-targeting sgRNA-encoding plasmid, single-guide RNAs were also designed and separately cloned into the plasmid LentiCRISPRV_2_Puro following the Zhang Lab protocol to generate the *NCL* and *PPIA* knock-out cell lines. To construct the dox-inducible bovine *TRIM5α* and *PPIA*-encoding plasmid, the cDNA-encoding bovine *TRIM5α* and *PPIA* were cloned into the linearized plasmid pLVX-TetOn-Puro vector separately with an infusion enzyme. The primers involved in the experiment are shown in Table 1.

### 2.2. Cell Culture

MDBK wild-type (MDBK-WT) cells (ATCC, CCL-22) were stored in our laboratory and 293T cells were purchased from ATCC. Both cell lines were cultured in Dulbecco’s Modified Eagle Medium (DMEM) supplemented with 10% fetal bovine serum and 1 × penicillin–streptomycin at 37 °C in 5% CO_2_.

### 2.3. siRNA-Mediated Gene Knockdown

To knockdown *TRIM5α*, MDBK or 293T cells were plated on a 48-well plate at 2 × 10^4^ per well. The following day, MDBK cells were transfected with siRNA targeting bovine *TRIM5α* (*5′*-AAG GAG UG CAA AUG UUG UCT T-*3′*) and scrambled negative control siRNA with Lipofectamine RNAiMAX (Thermo Fisher Scientific, Waltham, MA, USA Cat# 13778150) in Opti-MEM. At 48 h post-transfection, the cells were re-plated into a 24-well plate for infection with specified amounts of lentiviruses.

### 2.4. Generation of a Cas9-Inducible Expression MDBK Cell Line (MDBK-iCas9)

293T cells producing viral particles via co-transfection of packaging plasmids. 293T cells were co-transfected with a plasmid encoding Cas9, pLVX-TetOn-blasticidin, along with the psPAX2 packaging plasmid and pMD2.G, using a PEI transfection reagent in a ratio of 3:3:1. The supernatant, which contained the recombinant lentivirus, was then harvested to infect MDBK cells. Two days later, cells were selected in the presence of 6 μg/mL blasticidin S HCl (Beyotime, Shanghai, China Cat#ST018) for 7 days. Cas9 expression was confirmed by Western blotting.

### 2.5. Generation of a TRIM5α Knock-Out Cell Line (MDBK-iCas9^TRIM5α−/−^)

#### 2.5.1. Screening of sgRNA

293T cells were co-transfected with the LentiCRISPRV_2_EGFP plasmid encoding *TRIM5α*-sgRNA#1-3 and the bovine *TRIM5α*-encoding plasmid pCAGGS-HA-*TRIM5α* at a ratio of 1:2 using a PEI transfection reagent; the cell populations were collected at 48 h after transfection for Western blot identification. Expression of bovine *TRIM5α* was used as the marker to analyze the efficiency of sgRNAs. The primers involved in the experiment are shown in Table 1.

#### 2.5.2. Transfection of MDBK-iCas9 Cells and Single-Clone Selection

To generate the *TRIM5α* knock-out cell line, Cas9-overexpressing MDBK cells were seeded one day before transfection and subsequently transfected with the *TRIM5α*-targeting sgRNA-encoding LentiCRISPRV_2_EGFP plasmid according to the manufacturer’s instructions by using Lipofectamine 3000 (Thermo Fisher Scientific, Cat# L3000015). Transfected monoclonal MDBK cells expressing the EGFP protein were screened by flow cytometry at 48 h after transfection.

#### 2.5.3. DNA Extraction, PCR, and Sequencing

The DNA of each single clonal cell population was extracted by using the TIANamp Genomic DNA Kit (TIANGEN, Beijing China Cat# DP304-03) according to the manufacturer’s instructions. PCR was used to confirm the knock-out of *TRIM5α*; the 2 × Rapid Taq Master Mix (Vazyme, Nanjing, Jiangsu Province, China Cat# P222-03) in combination with *TRIM5α*-specific primers (primer forward: *5′*-CCT TGA TTT CTA GAT AAC TAG TG-*3′*; primer reverse: *5′*-GCC AGG TAA TTA AAG TCC AAT GCA-*3′*) was used for amplification. PCR products containing the target site were analyzed by gel electrophoresis, and bands were extracted from the gel with the GeneJET Gel Extraction Kit (Thermo Fisher Scientific, Cat# K0692) according to the manufacturer’s instructions. The PCR gel extract was further characterized by sequencing. The primers involved in the experiment are shown in Table 1.

#### 2.5.4. Western Blot Identification

The expression level of bovine TRIM5α in MDBK cells was confirmed by a Rabbit anti-TRIM5α polyclonal antibody, and HA-tagged TRIM5α in 293T cells was confirmed by an anti-HA Tag mouse mAb. The membrane was re-probed with a mouse anti-*β-actin* monoclonal antibody as a loading control. The bands were visualized using IRDye^®^ 680RD Goat anti-Mouse IgG Secondary Antibody and IRDye^®^ 800CW Goat anti-Rabbit IgG Secondary Antibody.

### 2.6. Generation of an NCL Knock-Out MDBK Cell Line

293T cells were co-transfected with a plasmid LentiCRISPRV_2_Puro encoding *NCL*-targeting sgRNA, along with the psPAX2 packaging plasmid and pMD2.G, using a PEI transfection reagent in a ratio of 3:3:1. The supernatant, which contained the recombinant lentivirus, was then harvested to infect MDBK-WT and MDBK-iCas9*^TRIM5α−/−^* cells. Two days later, cells were selected in the presence of 2 μg/mL puromycin (Invivogen, Carlsbad, CA, USA Cat# ant-pr-1) for 72 h; the total DNA of surviving cells was extracted and amplified with PCR. The PCR products were cloned into the pMD18-T vector and identified by sequencing. The primers involved in the experiment are shown in Table 1.

### 2.7. Generation of a PPIA Knock-Out MDBK Cell Line

293T cells were co-transfected with a plasmid LentiCRISPRV_2_Puro encoding bovine *PPIA*-targeting sgRNA, along with the psPAX2 packaging plasmid and pMD2.G, using a PEI transfection reagent in a ratio of 3:3:1. The supernatant, which contained the recombinant lentivirus, was then harvested to infect MDBK and MDBK-iCas9*^TRIM5α−/−^* cells. Two days later, cells were selected in the presence of 2 μg/mL of puromycin for 72 h, and surviving cells were then diluted into monoclonal cells. After 10 days of culture, the DNA of bovine *PPIA* of each monoclonal cell was extracted and identified by sequencing. The primers involved in the experiment are shown in Table 1.

### 2.8. Generation of a Bovine TRIM5α/PPIA-Inducible Expression 293T Cell Line

293T cells were co-transfected with a plasmid encoding bovine *TRIM5α* or *PPIA*, pLVX-TetOn-Puro, along with the psPAX2 packaging plasmid and pMD2.G, using a PEI transfection reagent in a ratio of 3:3:1. The supernatant, which contained the recombinant lentivirus, was then harvested to infect 293T cells. Two days later, cells were selected in the presence of 1.5 μg/mL of puromycin for 2 days. Bovine TRIM5α or CypA expression was separately confirmed by Western blotting. The primers involved in the experiment are shown in Table 1.

### 2.9. Quantification of mRNA via RT-PCR

To assess the mRNA levels in cells, total RNA was extracted using TRIzol™ Reagent (Thermo Fisher Scientific, Cat# 15596026). Subsequently, the RNA was subjected to reverse transcription using a PrimeScript™ RT reagent kit (Takara, Tokyo, Japan Cat# 2690C), followed by quantification of mRNA expression through RT-PCR utilizing the ChamQ Universal SYBR qPCR Master Mix (Vazyme, Cat#Q711-02). The primers involved in the experiment are shown in Table 1. The RT-PCR analysis was conducted using the QuantStudio Real-Time PCR System (Thermo Fisher Scientific), and the Ct values were normalized to the mean values obtained using *β-actin* as a housekeeping gene. The data were analyzed with the 2^−ΔΔCT^ method [21].

### 2.10. Statistical Analysis

Statistical analysis was performed using Graphpad Prism9.0 software, and the *t*-test was applied with a minimum of three independent replicates. A significance level of <0.05 was considered to indicate statistical significance for each test. Statistically significant differences between groups were indicated by * *p* < 0.1, ** *p* < 0.01, *** *p* < 0.001, and **** *p* < 0.0001.

## 3. Results

### 3.1. TRIM5α Limited Lentivirus Infection Efficiency in MDBK Cells

**Figure 1:**
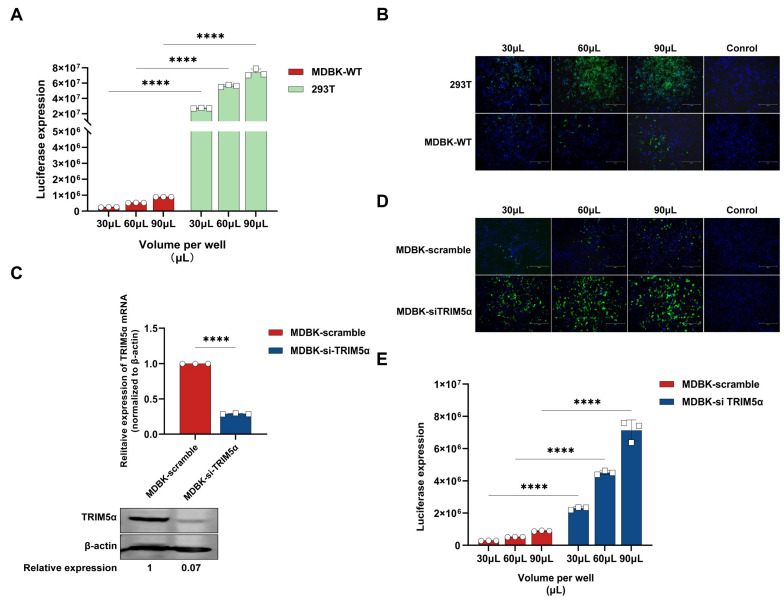
*TRIM5α* limited lentivirus infection efficiency in MDBK cells. (**A**) MDBK and 293T cells were infected with different amounts of HIV-1-based lentivirus encoding a luciferase reporter protein (30, 60, 90 μL). Luciferase expression was determined at 48 h post-infection. (**B**) MDBK and 293T cells were infected with different amounts of HIV-1-based lentivirus encoding an EGFP reporter protein (30, 60, 90 μL). EGFP (green), a surrogate marker of infection, and nuclei were stained with DAPI (blue) and observed with a fluorescence microscope. (**C**) RT-PCR and Western blotting were used to measure the mRNA and protein expression of *TRIM5α* in MDBK cells that were transfected with siRNA targeting bovine *TRIM5α* or scramble siRNA, and the relative protein expression level of TRIM5α was shown with densitometry. (**D**) MDBK cells transfected with siRNA targeting *TRIM5α* or scramble siRNA were infected with different amounts of HIV-1-based lentivirus encoding an EGFP reporter protein (30, 60, 90 μL). The fluorescence, a surrogate marker of infection, was observed with a fluorescence microscope at 48 h post-infection. (**E**) MDBK cells transfected with siRNA targeting *TRIM5α* or scramble siRNA were infected with different amounts of the HIV-1-based lentivirus encoding a luciferase reporter protein (30, 60, 90 μL). Luciferase expression was determined at 48 h post-infection. The results are presented as the mean and standard deviation of triplicate measurements from one assay, and they are representative of at least three independent experiments. Differences were examined with a two-tailed, unpaired Student’s *t*-test. **** *p* < 0.0001.

To verify the poor transduction of lentivirus in MDBK cells, different amounts of lentivirus encoding a luciferase or EGFP reporter protein were used to evaluate the difference in the infection efficiency between 293T cells and MDBK cells. As shown in Figure 1A, the expression of luciferase in 293T cells was at least 80-fold higher than that in MDBK cells. Similarly, the expression of EGFP in 293T cells was also significantly higher than that in MDBK cells (Figure 1B). These results demonstrate that, compared to 293T cells, lentivirus infection was limited in MDBK cells.

It has been proven that bovine *TRIM5α* can inhibit lentivirus replication in bovine cells [22]. To further identify whether bovine *TRIM5α* is a lentivirus restriction factor in MDBK cells, siRNA targeted to bovine *TRIM5α* was designed and transfected into MDBK cells, and the HIV-1-based lentivirus encoding a luciferase or EGFP reporter protein was used to evaluate infection efficiency in the *TRIM5α* knock-down MDBK cells. The mRNA and protein expression level of *TRIM5α* in MDBK cells transfected with siRNA was determined via quantitative real-time reverse transcription (RT-PCR) and Western blotting (Figure 1C). The results demonstrate that siRNA transfection decreased *TRIM5α* mRNA levels by 75% versus those in MDBK scramble cells and decreased TRIM5α protein levels by 93% versus those in MDBK scramble cells. As shown in Figure 1D, the expression levels of EGFP in *TRIM5α* knock-down cells were significantly increased compared to MDBK scramble cells; in the same way, the expression levels of luciferase show a 7–8-fold increase compared to MDBK scramble cells (Figure 1E). In conclusion, the lentivirus shows higher transduction efficiency in the *TRIM5α* knock-down MDBK cells, which indicates that *TRIM5α* plays an important inhibitory role in the process of lentivirus infection.

### 3.2. Generation of an Engineered MDBK Cell Line for Gene Editing

**Figure 2:**
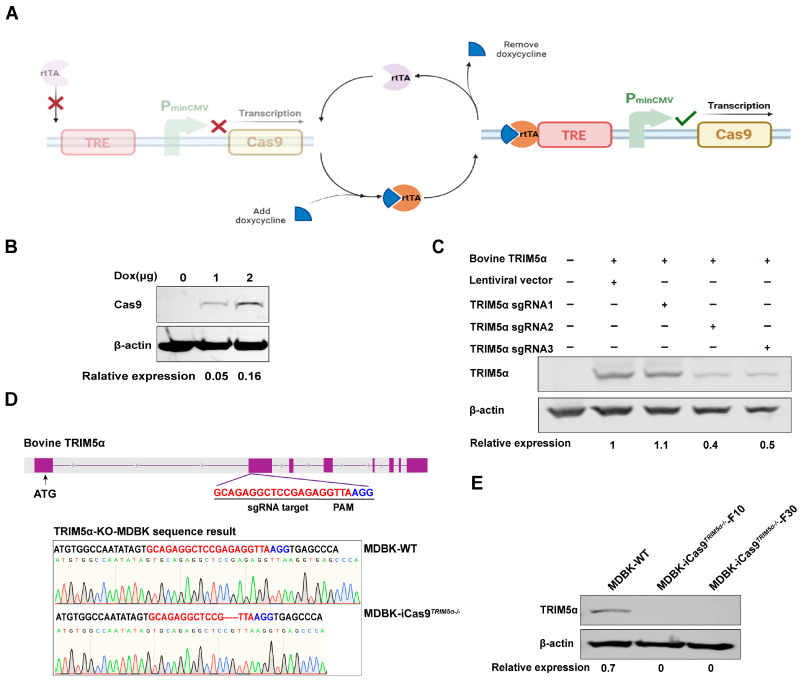
Generation of an engineered MDBK cell line for gene editing. (**A**) The figure shows the schematic representation of the dox-inducible Cas9 overexpression system. (**B**) Western blot detection of Cas9 protein expression in dox-induced Cas9-overexpressing 293T cells, and the relative protein expression level of Cas9 is shown with densitometry. (**C**) The *TRIM5α* encoding plasmid and sgRNA#1-3-encoding plasmids were co-transfected into 293T cells, and TRIM5α expression was identified by Western blotting to determine sgRNA cleavage efficiency. The cellular lysate of 293T cells co-transfected with the *TRIM5α*-encoding plasmid and the empty lentivirus vector was used as a positive control, and the cellular lysate of unmodified 293T cells was used as a negative control. The relative protein expression level of TRIM5α is shown with densitometry. (**D**) A schematic representation of the sgRNA target site on the *TRIM5α* gene. The genomic DNA of *TRIM5α* knock-out cells was amplified using PCR, and the PCR product was subjected to sequencing to identify gene editing. The sequencing results are displayed in the lower portion of the image. (**E**) Western blot detection of the expression of the TRIM5α protein in MDBK-WT cells and different generations of MDBK-iCas9*^TRIM5α−/−^* cells. *β-actin* was used as a housekeeping protein, and the relative protein expression level of TRIM5α is shown with densitometry.

Based on the finding that *TRIM5α* plays a restricted role in lentivirus infection, we next focused on a method for enhancing the gene editing capabilities of MDBK cells. First, a doxycycline(dox)-inducible Cas9-overexpressing MDBK cell line was constructed through the Tet-On system (Figure 2A) to improve the subsequent CRISPR/Cas9-based gene editing efficiency. MDBK cells were infected with the Cas9-encoding lentivirus for 48 h and selected with blasticidin at a concentration of 6 μg/mL for about a week. The surviving cells were treated with different concentrations of dox to verify Cas9 protein expression. The Western blot result of Figure 2B identifies that Cas9 protein expression increases in a dox-dependent manner. The cell line was defined as an inducible Cas9-overexpressing MDBK cell line (MDBK-iCas9).

Based on the result that Cas9 overexpression could enhance the CRISPR/Cas9 system’s gene editing efficiency, we next knocked out the *TRIM5α* of the MDBK-iCas9 cell line to increase lentivirus transduction efficiency. Three single-guide RNAs (sgRNAs) targeting bovine *TRIM5α* exon2 were designed and cloned into the LentiCRISPRV_2_EGFP vector to generate an sgRNA-expressing plasmid, LentiCRISPRV_2_EGFP-*TRIM5α*-sgRNA#1-3. First, the sgRNAs were screened according to cleavage efficiency to improve gene editing efficiency. Then, the plasmid LentiCRISPRV_2_EGFP-*TRIM5α*-sgRNA#1-3 was co-transfected with an HA-tagged bovine *TRIM5α*-encoding plasmid into 293T cells, and the expression of TRIM5α was measured by Western blotting to compare the cleavage efficiency between the three sgRNAs (Figure 2C). Through the densitometry of the Western blot results, we demonstrate that sgRNA#2 has the best cleavage efficiency. Next, the plasmid LentiCRISPRV_2_EGFP-*TRIM5α*-sgRNA#2 was transfected into the MDBK-iCas9 cell line, and monoclonal MDBK cells expressing the EGFP protein were screened by flow cytometry at 48 h post-transfection. After 10 days of culture, DNA from each monoclonal cell was extracted, and the *TRIM5α* fragment was amplified by PCR with primers flanking the targeting site and analyzed by sequencing. The sequencing results show that one cell clone harbored gene editing at the targeted site and caused a 5bp deletion (Figure 2D). Based on the sequencing data, the selected clone was next identified by Western blotting to evaluate the level of bovine TRIM5α expression. Based on the result of the Western blot (Figure 2E), we observed no detectable expression of the TRIM5α protein in the *TRIM5α* knock-out MDBK cell line. The cell clone was defined as MDBK-iCas9*^TRIM5α−/−^*. To summarize the above results, the MDBK-iCas9*^TRIM5α−/−^* cell line was successfully constructed.

### 3.3. The MDBK-iCas9^TRIM5α−/−^ Cell Line Exhibits Enhanced Capability for Gene Editing

**Figure 3:**
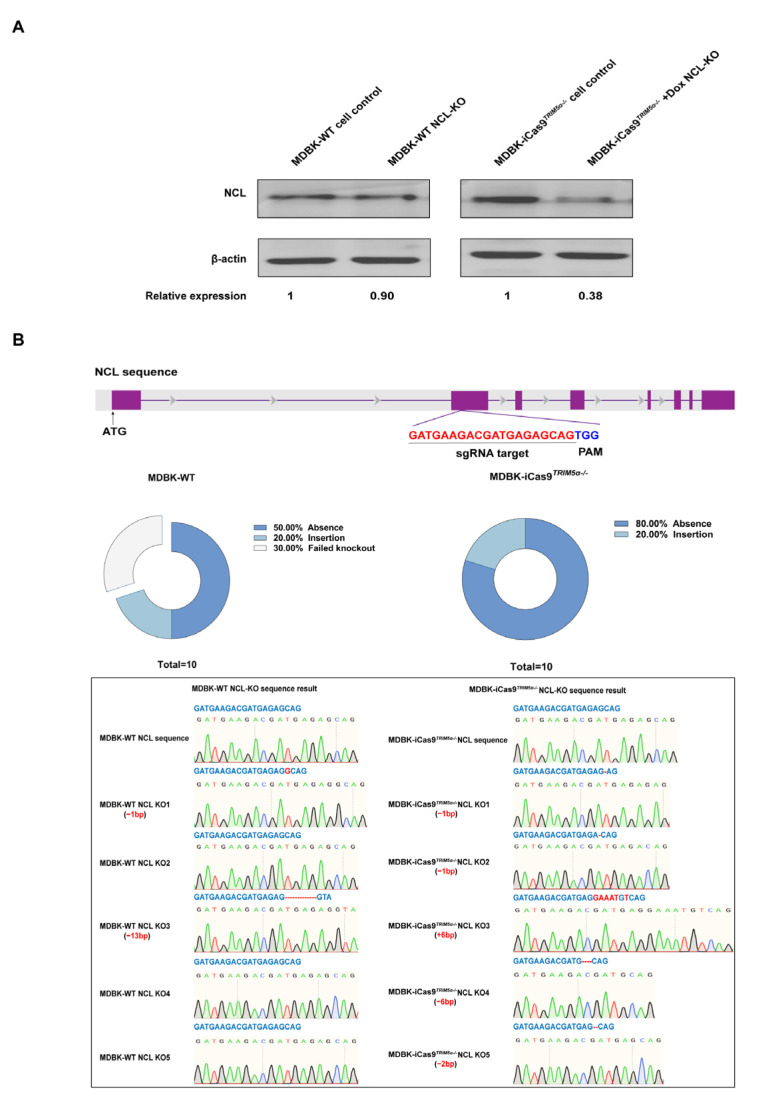
The MDBK-iCas9*^TRIM5α−/−^* cell line exhibits enhanced capability for gene editing. (**A**) Western blotting was used to identify *NCL* expression in the MDBK-WT-based *NCL* knock-out cell line and dox-treated/untreated MDBK-iCas9*^TRIM5α−/−^*-based *NCL* knock-out cell line. The MDBK-WT cell line was used as a positive control, *β-actin* was used as a housekeeping protein, and the relative protein expression level of *NCL* is shown with densitometry. The surviving cells of the MDBK-WT-based *NCL* knock-out cell line and the dox-treated MDBK-iCas9*^TRIM5α−/−^*-based *NCL* knock-out cell line after 72 h of adding puromycin. (**B**) A schematic representation of the sgRNA target site on *NCL*. The genomic DNA of the MDBK-iCas9*^TRIM5α−/−^* cell line and MDBK-WT-based *NCL* knock-out cells was amplified using PCR, and the PCR product was cloned into a pMD 18-T vector, with 10 single clones of each cell line selected for sequencing. The sequencing results and statistical analysis of knock-out effectiveness are shown.

To evaluate the gene editing efficiency of the MDBK-iCas9*^TRIM5α−/−^* cell line, we knocked out the gene *Nucleolin* (*NCL*), which is under investigation in our laboratory, in the MDBK-WT cell line and MDBK-iCas9*^TRIM5α−/−^* cell line by using the lentivirus-based CRISPR/Cas9 system. We next knocked out *NCL* in the MDBK cell line and MDBK-iCas9*^TRIM5α−/−^* cell line to compare the gene editing efficiency of the two cell lines. The MDBK-iCas9*^TRIM5α−/−^* cell line was pretreated with dox to induce the expression of the protein Cas9, and the cells of both cell lines were selected by puromycin (2 μg/mL) for 72 h. The surviving cells of the *NCL*-KO cell line, based on the MDBK-WT and MDBK-iCas9*^TRIM5α−/−^* cell lines, were collected for Western blotting to compare the efficiency of lentivirus infection by identifying the expression of *NCL*. Based on the result of Western blotting (Figure 3A), compared to the MDBK-WT cell line, the expression of *NCL* in the MDBK-based *NCL*-KO cell line exhibited a 10% decrease, but compared to the MDBK-iCas9*^TRIM5α−/−^* cell line, the expression of *NCL* in the dox-treated MDBK-iCas9*^TRIM5α−/−^* cell line exhibited a 62% decrease, which indicates that the gene editing efficiency of MDBK can be substantially enhanced by combining *TRIM5α* knock-out with overexpression of Cas9 following dox treatment. At the same time, the DNA of surviving cells in the MDBK-iCas9*^TRIM5α−/−^*-based *NCL*-KO cell line and MDBK-based *NCL*-KO cell line was also extracted and amplified by PCR with primers flanking the target site, and the PCR product was then purified and cloned into a pMD 18-T vector. A total of 10 monoclonal cells were selected from the product of each cell line ligation vector for sequencing to compare knock-out efficiency. The sequence result shows that the gene editing efficiency of the dox-treated MDBK-iCas9*^TRIM5α−/−^* cell line was 100%, while the efficiency of the MDBK-WT cell line was 70% (Figure 3B), which indicates that the MDBK-iCas9*^TRIM5α−/−^* cell line is more suitable for gene editing.

### 3.4. The Absence of TRIM5α and the Addition of CsA Can Independently Exert a Lentivirus-Promoting Effect in MDBK Cells

**Figure 4:**
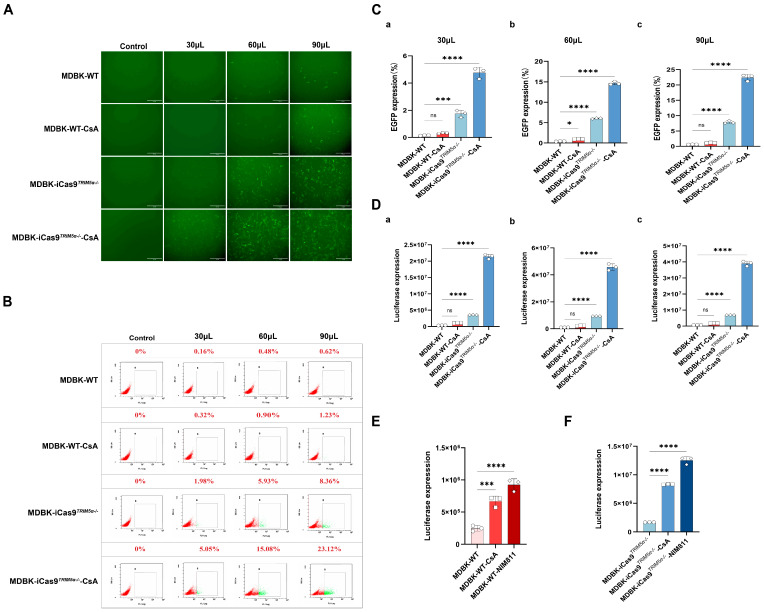
The absence of *TRIM5α* and the addition of CsA can independently exert an HIV-1-based lentivirus-promoting effect in MDBK cells. (**A**) The MDBK-iCas9*^TRIM5α−/−^* cell line and the MDBK-WT cell line were infected with different volumes of HIV-1-based lentivirus encoding an EGFP reporter protein (30, 60, 90 μL) in the presence or absence of CsA (2 µM). EGFP (green), a surrogate marker of infection, was observed with a fluorescence microscope and (**B**) measured using a flow cytometer at 48 h post-infection. (**C**) Statistical analysis of the results of flow cytometry. (**D**) The MDBK-WT cell line and the MDBK-iCas9*^TRIM5α−/−^* cell line were infected with different volumes of HIV-1-based lentivirus encoding a luciferase reporter protein ((**a**) 30, (**b**) 60, (**c**) 90 μL) in the presence or absence of CsA (2 µM). Luciferase expression was determined at 48 h post-infection. (**E**) The MDBK-WT cell line was infected with HIV-1-based lentivirus encoding a luciferase reporter protein (30 µL) in the presence of CsA or NIM811 (8 µM). Luciferase expression was determined at 48 h post-infection. (**F**) The MDBK-iCas9*^TRIM5α−/−^* cell line was infected with HIV-1-based lentivirus encoding a luciferase reporter protein (30 µL) in the presence of CsA or NIM811 (8 µM). Luciferase expression was determined at 48 h post-infection. Statistically significant differences between groups were indicated by * *p* < 0.1, *** *p* < 0.001, and **** *p* < 0.0001.

To further quantify the enhanced HIV-1-based lentivirus transduction efficiency of the MDBK-iCas9*^TRIM5α−/−^* cell line, the HIV-1-based lentivirus encoding a luciferase or EGFP reporter protein was used to evaluate the impact of *TRIM5α* knock-out. The results of the fluorescent picture in Figure 4A and the flow cytometry in Figure 4B represent the expression level of EGFP after lentivirus infection at different volumes (30, 60, 90 μL). In combination with the statistical analysis of the flow cytometry results (Figure 4C), the absence of *TRIM5α* leads to a 12-fold increase in EGFP expression and a 9–10-fold increase in luciferase expression, as shown in Figure 4D.

Previous studies have shown that the chemical compound cyclosporine A (CsA) can enhance the infection of the HIV-1 virus in new world monkey cells [18] but inhibit HIV-1 in human cells [16]. Similarly, there is research that has demonstrated that CsA can enhance the sensitivity of MDBK cells to HIV-1 [22]. Our work confirms these findings, and we also provide additional critical observations on the role of CsA and CypA in bovine cells. To further confirm the effects of CsA and explore its function in bovine cells, we next infected the MDBK-WT cell line and MDBK-iCas9*^TRIM5α−/−^* cell line with the HIV-1-based lentivirus encoding a luciferase or EGFP reporter protein in the presence of 2 µM CsA. The results of the fluorescent picture in Figure 4A and the flow cytometry in Figure 4B represent the expression level of EGFP after lentivirus infection at different volumes (30, 60, 90 μL). In combination with the statistical analysis of the flow cytometry results at 48 h post-infection (Figure 4C), CsA treatment increased the EGFP expression level of both the MDBK-WT cell line and MDBK-iCas9*^TRIM5α−/−^* cell line by 2–3 times. The results of luciferase expression are shown in Figure 4D. CsA treatment increased luciferase expression about two to three times in the MDBK-WT cell line but enhanced luciferase expression in the MDBK-iCas9*^TRIM5α−/−^* cell line about four to five times. Interestingly, while CsA’s function was previously established as TRIM5α-dependent in human and new world monkey cells, our findings in bovine cells reveal a distinct mechanism. Specifically, the CsA-treated TRIM5α knock-out MDBK cell line (MDBK-iCas9*^TRIM5α−/−^*) exhibited higher lentiviral transduction efficiency compared to wild-type MDBK cells (MDBK-WT). This suggests that CsA’s activity in bovine cells operates independently of TRIM5α.

The chemical compound CsA can interact with the cell protein cyclophilin A (CypA) [23], hindering the replication of HIV-1. At the same time, the CypA-CsA complex evokes an immunosuppressive effect, inhibiting the activity of immune cells, which could potentially suppress HIV-specific immune responses [24]. To determine which method CsA used to promote lentivirus in bovine cells, the non-immunosuppressive CsA derivative NIM811 [25,26] was used to pretreat cells in lentivirus infection experiments. The MDBK-WT cell line (Figure 4E) and MDBK-iCas9*^TRIM5α−/−^* cell line (Figure 4F) were infected with the same volume of the HIV-1-based lentivirus encoding a luciferase reporter protein in the presence of 2 µM CsA or 8 µM NIM811. Following treatment with NIM811, luciferase expression of the NIM811-treated MDBK-WT cells exhibited a two- to four-fold increase, while CsA-treated MDBK-WT cells exhibited a two- to three-fold increase. The NIM811-treated MDBK-iCas9*^TRIM5α−/−^* cell line exhibited a five- to seven-fold increase, while the CsA-treated MDBK-iCas9*^TRIM5α−/−^* cell line exhibited a four- to five-fold increase. These results show that both treatment with CsA and NIM811 could increase the expression of luciferase, indicating that the functions of CsA and NIM811 are independent of *TRIM5α* in bovine cells. Moreover, the results also demonstrate that the HIV-1-based lentivirus-promoting effect of CsA is exerted via interaction with CypA, and not immunosuppression.

Previous studies have demonstrated that the interaction target of CsA is the cell protein cyclophilin A (CypA) [23], which is translated by the gene *PPIA* and plays different roles in HIV-1 across different cell types. In human cells, CypA enhances HIV-1 infection by stabilizing the capsid protein of HIV-1 [23,27]. CsA treatment inhibits HIV-1 by competitively binding with CypA, but in new world monkey cells; CypA replaces the SPRY domain of TRIM5α and forms a TRIM5-CypA fusion protein, which strongly restricts HIV-1 via a more specific interaction with the capsid protein of HIV-1. CsA treatment enhances HIV-1 infection by competitively interacting with CypA [28]. However, in bovine cells, the structure and function of CypA are still unknown.

### 3.5. CypA Functions as a Lentivirus Restriction Factor Independently of TRIM5α in Bovine Cells

**Figure 5:**
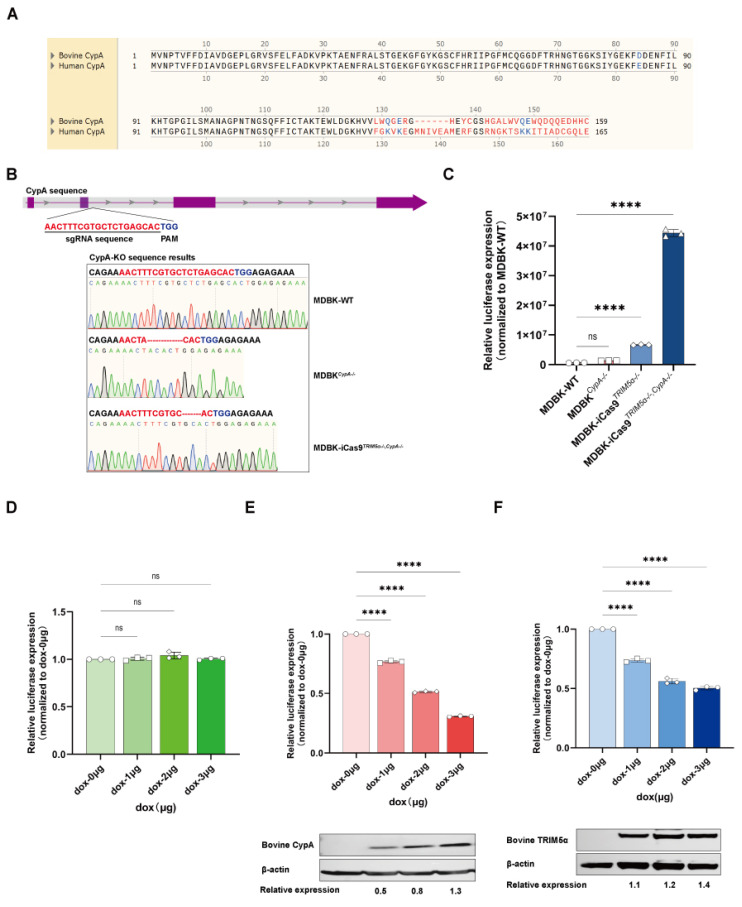
CypA functions as a lentivirus restriction factor independently of *TRIM5α* in bovine cells. (**A**) The protein sequences of bovine CypA and human CypA were compared. (**B**) A schematic representation of the sgRNA target site on the *PPIA* gene. The genomic DNA of *PPIA* Western blot cells was amplified using PCR, and the PCR product was subjected to sequencing to identify gene editing efficiency. The sequencing results are displayed in the lower portion of the image. (**C**) The MDBK-WT, MDBK*^CypA−/−^*, MDBK-iCas9*^TRIM5α−/−^*, and MDBK-iCas9*^TRIM5α−/−, CypA−/−^*cell lines were infected with HIV-1-based lentivirus encoding a luciferase reporter protein, and luciferase expression was determined at 48 h post-infection. (**D**) The 293T cell line was treated with dox at concentrations of 1, 2, and 3 μg/mL for 24 h and infected with 60 µL of HIV-1-based lentivirus encoding a luciferase reporter protein, with luciferase expression determined at 48 h post-infection. (**E**) The dox-inducible bovine *PPIA*-overexpressing 293T cell line was treated with dox at concentrations of 1, 2, and 3 μg/mL for 24 h and infected with 60 µL HIV-1-based lentivirus encoding a luciferase reporter protein, with luciferase expression determined at 48 h post-infection. The expression of bovine CypA was detected with Western blotting, and the relative protein expression level of bovine CypA is shown with densitometry. (**F**) The dox-inducible bovine *TRIM5α*-overexpressing 293T cell line was treated with dox at concentrations of 1, 2, and 3 μg/mL for 24 h and infected with 60 µL HIV-1-based lentivirus encoding a luciferase reporter protein, with luciferase expression determined at 48 h post-infection. The expression of bovine TRIM5α was detected with Western blotting, and the relative protein expression level of bovine TRIM5α is shown with densitometry. Statistically significant differences between groups were indicated by **** *p* < 0.0001.

To investigate whether bovine CypA plays a different function from human CypA, the protein sequences of bovine CypA and human CypA were compared (Figure 5A). The initial 128-amino-acid sequences of both CypA proteins are identical. However, the sequence of the two CypA proteins encodes distinct proteins from the 129th amino acid, which may lead to different functions. To further demonstrate the function of bovine CypA independently of *TRIM5α*, we next knocked out *PPIA* of the MDBK cell line and MDBK-iCas9*^TRIM5α−/−^* cell line by using a lentivirus-based gene editing method to compare the susceptibility of MDBK cells to HIV-1-based lentivirus following *PPIA* knock-out. The sequencing results are shown in Figure 5B. The *PPIA* of MDBK-WT shows a 13bp deletion and 1bp insertion, and the *PPIA* of MDBK-iCas9*^TRIM5α−/−^* shows a 7bp deletion. Altogether, the cell clone of the *PPIA* knock-out MDBK cell line was defined as MDBK*^CypA−/−^*, and the *PPIA* knock-out MDBK-iCas9*^TRIM5α−/−^* cell line was defined as MDBK-iCas9*^TRIM5α−/−, CypA−/−^*. The HIV-1-based lentivirus encoding a luciferase reporter protein was used to infect MDBK-WT, MDBK*^CypA−/−^*, MDBK-iCas9*^TRIM5α−/−^*, and MDBK-iCas9*^TRIM5α−/−, CypA−/−^*cells (Figure 5C). Similar to the results of the CsA and NIM811-treated MDBK and MDBK-iCas9*^TRIM5α−/−^*cells, the expression of luciferase shows a 1.5–2-fold increase in the MDBK*^CypA−/−^*cell line compared to MDBK-WT cells but a 5–8-fold increase in MDBK-iCas9*^TRIM5α−/−, CypA−/−^*cells compared to MDBK-iCas9*^TRIM5α−/−^* cells, which indicates that the function of CypA is not dependent on *TRIM5α* but rather exhibits enhanced anti-lentivirus activity in the absence of *TRIM5α* expression. To further evaluate the anti-lentivirus function of bovine CypA, we next generated the dox-inducible bovine *PPIA* and *TRIM5α*-overexpressing 293T cell lines. The 293T cells (Figure 5D) and dox-inducible bovine *PPIA* and *TRIM5α*-overexpressing 293T cell lines (Figure 5E,F) were treated with dox at a concentration of 1–3 μg/mL for 24 h and infected with HIV-1-based lentivirus encoding a luciferase reporter protein. The cell lysis was collected at 48 h post-infection for luciferase expression detection and CypA or TRIM5α expression detection. As shown in the results, the expression of bovine CypA and TRIM5α increased as the concentration of dox increased, and luciferase expression decreased as the expression of CypA and TRIM5α increased, which means that both bovine CypA and TRIM5α can inhibit lentivirus independently. To summarize, bovine CypA functions as an HIV-1-based lentivirus restriction factor independently of *TRIM5α* in bovine cells.

## 4. Discussion

In this study, we exploited two methods to improve the efficiency of HIV-1-based gene editing in MDBK cells: CRISPR/Cas9 gene editing efficiency targeting and cytokine targeting. Firstly, we enhanced CRISPR/Cas9 gene editing efficiency in MDBK cells via Cas9 protein overexpression and sgRNA screening. The expression of Cas9 plays an important role in the whole gene editing process. Thus, we generated the inducible Cas9-overexpressing MDBK cell line (MDBK-iCas9) with the lentivirus-based method. The inducible overexpression of the protein Cas9 can improve gene editing efficiency while maintaining the cell lines’ normal proliferation activities. The efficiency of sgRNA cleavage and the probability of off-target effects influence gene editing. Therefore, we used the protein expression level as a marker to screen the sgRNAs. The sgRNAs were co-transfected with the gene encoding the target protein into 293T cells, and the expression of the target protein was used as an index for evaluating the cleavage efficiency of sgRNAs. The most efficient sgRNA was determined in this experiment by comparing the expression of bovine TRIM5α. Notably, various cell types exhibit high lentiviral transduction efficiency, but there still exist issues with low Cas9 protein expression and off-target effects of sgRNA, which limit the efficiency of gene editing [29,30]. Therefore, Cas9 overexpression and sgRNA screening by cleavage efficiency are efficient ways to improve gene editing, which are suitable for both transfection and lentivirus transduction gene editing.

Secondly, we focused on cell targeting to increase the gene editing efficiency of lentivirus in MDBK cells. After identifying the anti-lentivirus vector function of *TRIM5α* in MDBK cells by siRNA transfection, we knocked out *TRIM5α* in the MDBK-iCas9 cell line with the transfection method, which avoids the insertion of foreign genes. Combining the sequencing and Western blot results, the MDBK-iCas9*^TRIM5α−/−^* cell line was constructed. The absence of *TRIM5α* significantly elevated the susceptibility of MDBK cells to lentivirus. To further enhance lentiviral transduction efficiency, we treated the TRIM5α knock-out MDBK cell line (MDBK-iCas9*^TRIM5α−/−^*) with cyclosporine A (CsA). Remarkably, CsA increased HIV-1-based lentiviral infection regardless of *TRIM5α* expression status—a finding in contrast to observations in human and new world monkey cells, where CsA’s activity is TRIM5α-dependent [31]. To eliminate potential immunosuppressive effects attributed to CsA, we utilized NIM811, a non-immunosuppressive derivative of CsA [25], which remains immune pathway-neutral compared to CsA. The luciferase expression of HIV-1-based lentivirus exhibited similar trends in NIM811-treated MDBK cells to CsA, showing that CsA in bovine cells mainly functions through its combination with CypA, further facilitating the replication of lentivirus. Altogether, treatment with CsA or NIM811 could promote lentivirus infection via interaction with bovine CypA. In conclusion, combining *TRIM5α* knock-out and CsA or NIM811 treatment can work as an effective cell targeting strategy to significantly improve HIV-1-based lentivirus transduction efficiency in MDBK cells.

Given the shared involvement of TRIM5 and CypA in HIV-1 infectivity, these host factors have been historically co-investigated in retroviral restriction studies. The cell factor TRIM5α can directly interact with the HIV-1 capsid protein CA and induce premature or non-productive uncoating that disrupts reverse transcription [32,33]. Previous research showed that the SPRY domain of TRIM5α recognizes and interacts with the CA protein [5], thus enabling TRIM5α to exert its anti-HIV function. In our study, we found the function of bovine CypA to be different from that of human or new world monkey CypA, as reported in other experiments [16,18]. In human cells, CypA protects HIV-1 from the CA-specific restriction factor TRIM5α by competitively binding with CA. This interaction not only inhibits the conjunction of TRIM5α and CA but also enhances the stability of the HIV capsid protein to facilitate HIV replication. Disruption of CA-CypA binding by CsA decreases HIV-1 infectivity in human cells [34,35,36,37]. However, in new world monkey cells, CypA replaced parts of the domain of the TRIM5α protein, forming a new protein, TRIM5-CypA [17,18,38]. The fusion protein TIRM5-CypA was found to restrict HIV-1 replication due to the higher-affinity interaction of TRIM5-CypA and CA, which enhanced the anti-HIV functionality of the TRIM5 protein. Disruption of CA-CypA binding stimulates HIV infectivity in new world monkey cells [39]. In this study, we explored the function of CypA in bovine cells. Our study shows that the CypA-targeting compound can increase the HIV-1-based lentivirus transduction efficiency in both the MDBK-WT and MDBK-iCas9*^TRIM5α−/−^* cell lines, and this function seems to be independent of whether TRIM5α can be expressed normally, which is different from CypA in human and new world monkey cells. Could this discrepancy be attributed to the structural or functional differences in CypA between human and bovine cells?

Like human *PPIA*, bovine *PPIA* forms an independent open reading frame. Bovine CypA comprises 159 amino acids (NP_847890.1), while human CypA has 165 amino acids (NP_066953.1). In the protein domain analysis from NCBI, bovine CypA and human CypA share almost the same first 128-amino-acid sequence, differing from the 129th amino acid onwards. However, these two types of CypA exhibit contrasting functions during the lentivirus infection process. Interestingly, the 1–128 amino acids of bovine CypA have been defined as cyclophilin-type peptidylprolyl cis-trans isomerases, which exhibit peptidylprolyl cis-trans isomerase activity (*PPIase*, *Rotamase*) and bind to the chemical compound CsA. Yet the 129–159 amino acids of bovine CypA remain uncharacterized, probably resulting in the functional divergence between the species of CypA. Our study found that bovine CypA functions as an HIV-1-based lentivirus restrictor in bovine cells regardless of TRIM5α expression, demonstrating that bovine CypA can independently exert an anti-HIV-1 effect. It is worth noting that the anti-HIV function of CypA in cell lines with normal TRIM5α expression, such as the MDBK cell line, is significantly weaker than that in the *TRIM5α* knock-out cell line. In other words, in bovine cells, *TRIM5α* likely serves as a primary anti-HIV-1 factor, and its normal expression likely suppresses the antiviral function of CypA. CypA may assume an anti-HIV-1 role when TRIM5α is either not expressed normally or not functioning normally. Thus, research on the target of bovine CypA on HIV-1-based lentivirus and its anti-lentivirus mechanism can inform the approach of subsequent anti-HIV strategies. However, this study employed only the modified HIV-1-based lentivirus as a model. The inhibitory effect of bovine CypA on other lentiviruses requires further validation.

In conclusion, we found that both bovine CypA and *TRIM5α* function as an HIV-1 inhibitor in MDBK cells, which indicates that the MDBK-iCas9*^TRIM5α−/−^* cell line, combining the CypA binding chemical compound CsA could be used as a more convenient gene editing tool for in vitro investigations of virus–host protein interactions.

## Figures and Tables

**Table 1:** The primers involved in the experiment.

Plasmids or Primers	Primers or Probes	Sequence (5′-3′)
pLVX-TetOn-Blast	pLVX-TetOn-blast-F	TGAGGAGGCTTTTTTGGAGGCCTAGGCTTTTGCAAAACGCGACCATGGCCAAGCCTTTGTCTCAAGAAG
	pLVX-TetOn-blast-R	CTTTTCACAAATTTTGTAATCCAGAGGTTGATTGTTCCAGACGCGTTTAGCCCTCCCACACATAAC
pLVX-TetOn-Blast-Cas9	pLVX-TetOn-Blast-Cas9-F	TCCTACCCTCGTAAGGAATTCGCCACCATGGACAAGAAGT
	pLVX-TetOn-Blast-Cas9-R	CAGGGGAGGTGGTCTGGATCCTCACTTATCGTGATCGTCTTTGTAAT
pLVX-TetOn-Blast-bovine-*TRIM5α*	pLVX-TetOn-*TRIM5α*-F	TCCTACCCTCGTAAGGAATTCTACCCATACGATGTTCCAGATTACGCT
	pLVX-TetOn-*TRIM5α*-R	TCGCAGGGGAGGTGGTCTGGATCCTCAACAGCTTGGTGAGCACAGAGT
pCAGGS-*TRIM5α*	pCAGGS-*TRIM5α*-F	CAGATTACGCTGAATTCATGGCTTC
	pCAGGS-*TRIM5α*R	CTGCTAGCTCGAGTCAACAGCTTGG
Bovine *TRIM5α* sgRNA	B-*TRIM5α* sgRNA#1	TCGGAGCCTCTGCACTATAT
Bovine *TRIM5α* sgRNA	B-*TRIM5α* sgRNA#2	GCAGAGGCTCCGAGAGGTTA
Bovine *TRIM5α* sgRNA	B-*TRIM5α* sgRNA#3	AGTGATACAGGCTTGACAGA
Bovine *NCL* sgRNA	B-*NCL* sgRNA	GATGAAGACGATGAGAGCAG
pLVX-TetOn-Blast-bovine-*PPIA*	pLVX-TetOn-*PPIA*-F	CCCTCGTAAGGAATTCATGGTCAACCCCACCGTG
	pLVX-TetOn-*PPIA*-R	GAGGTGGTCTGGATCCTCACTTATCATCGTCGTCCTTATAATCGATGTCGTGATCCTTGTAGTCCCCGTCGTGGTCCTTGTAGTCGATTTGTCCACAGTCA
Bovine *PPIA* sgRNA	B-*PPIA* sgRNA	AACTTTCGTGCTCTGAGCAC
Bovine *TRIM5α* mRNA	B-*TRIM5α*-F	ATCCAATGACCAACGCTC
	B-*TRIM5α*-R	TTTTCACCACATACCCCC
Bovine *β-actin* mRNA	B-*β-actin*-F	TGCTTCTAGGCGGACTGTTAG
	B-*β-actin*-R	CGCAAGTTAGGTTTTGTCAAGA
Bovine *TRIM5α*-KO identification	B-*TRIM5α*-KO identification-F	TCTTCACTTTTAACCTTTCCAATCATTCAGGGATCTGTGAGCGA
	B-*TRIM5α*-KO identification-R	CGAAGAAAAGAGATACAGGCCTCAAGTCCCTG
Bovine *NCL*-KO identification	B-*NCL*-KO identification-F	TCCATGGAATTCTCTAGGCAAGAATA
	B-*NCL*-KO identification-R	GGGAAGCTAGAAGAATCTGATAAGG
Bovine *PPIA*-KO identification	B-*PPIA*-KO identification-F	GACAAGGGTACTAAGCAACA
	B-*PPIA*-KO identification-F	ACTAGAAGGTCACTTGGAAC

## Data Availability

The data presented in this study are available upon request from the corresponding authors.
